# Gene Expression Profile of Gm20594 and Hapln1 in Mouse Cardiac Muscle Exposed to Microgravity and Space-like Chronic Low-Dose Radiation

**DOI:** 10.17912/micropub.biology.001746

**Published:** 2025-11-05

**Authors:** Emma Mackey, Carly Orr, Azemat Jamshidi-Parsian, Stephanie Byrum, Robert Griffin, Rupak Pathak, Nathan S. Reyna

**Affiliations:** 1 Biology, Ouachita Baptist University, Arkadelphia, Arkansas, United States; 2 University of Arkansas for Medical Sciences, Little Rock, Arkansas, United States; 3 Center for Proteomics and Metabolomics, St. Jude Children's Research Hospital, Memphis, Tennessee, United States; 4 Division of Radiation Health, University of Arkansas for Medical Sciences, Little Rock, Arkansas, United States; 5 Department of Pharmaceutical Sciences, University of Arkansas for Medical Sciences, Little Rock, Arkansas, United States

## Abstract

Exposure to cosmic radiation and microgravity has led to adverse health effects, including cardiac dysfunction, in astronauts after spaceflight. To better understand the underlying mechanisms, we concurrently exposed C57BL/6J mice to chronic irradiation and simulated microgravity for 30 days and collected cardiac tissue for gene expression profiling. Gm20594 and Hapln1 were found to be significantly differentiated in mouse cardiac muscle exposed to simulated microgravity and chronic irradiation. Existing literature detailing the typical function of these genes indicates that their differential expression can lead to heightened cardiovascular risk due to heart inflammation, reduced contractility, and increased apoptosis.

**Figure 1. Identification of target genes and Differential Gene Expression GM20594 and Hapln1:**
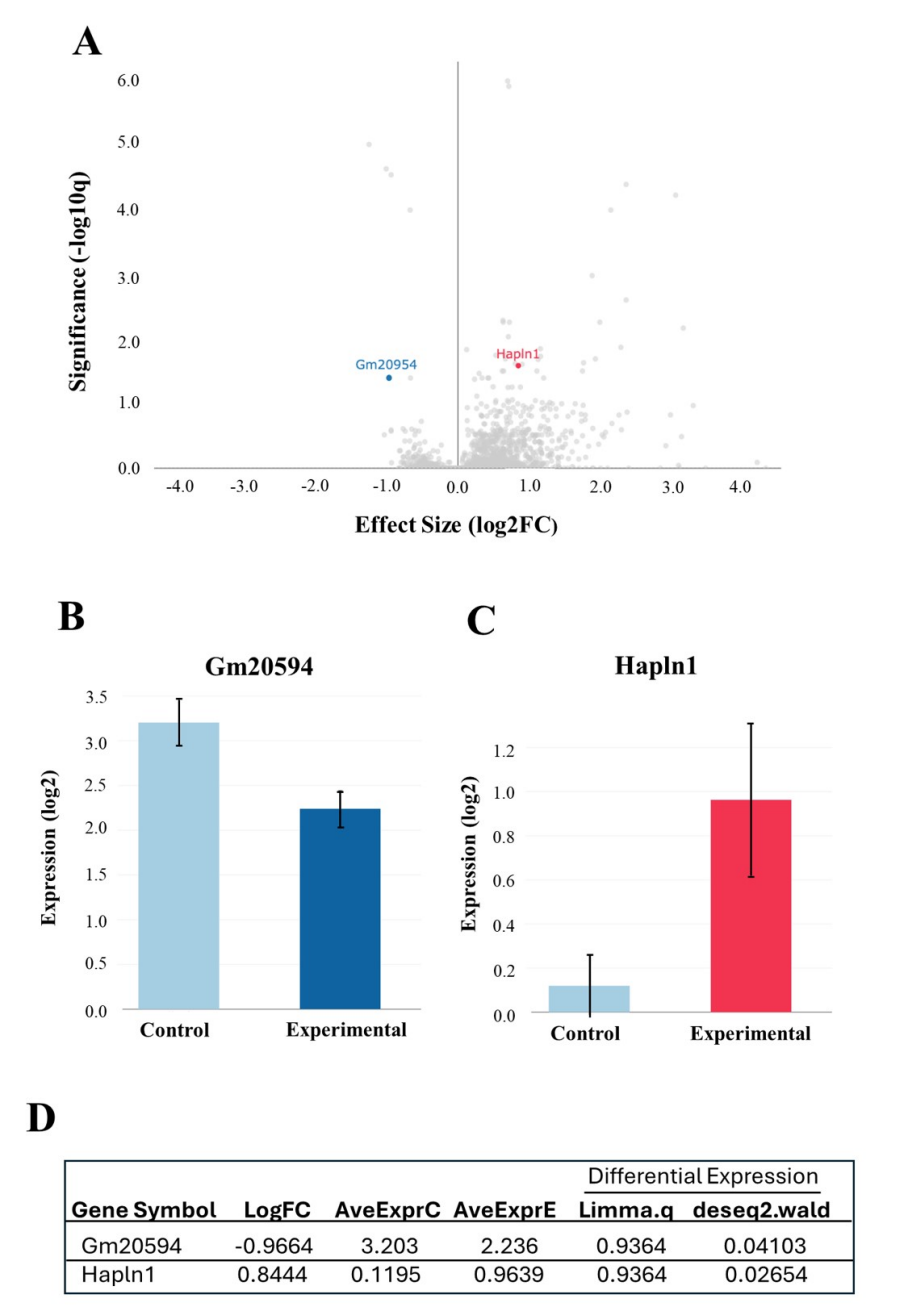
**
A: *Volcano Plot of Differential Gene Expression.*** Gm20594 (blue) was significantly downregulated (false discovery rate (FDR)<0.05, [Log2FC]=-0.97, [-Log10q] p=0.03). Hapln1 (red) was significantly upregulated (FDR<0.05, [Log2FC]=0.84, [-Log10q] p=0.03). While the degrees of freedom (df) is 6, the effect sizes being high indicates significant differential expression. **B: *Gm20594 Differential Expression Bar Plot.*** A DESeq2 Wald test was used to analyze the strength of differentiated genes Gm20594 and Hapln1 instead of solely noting the presence of differentiated genes (Love, *et al* ., 2014). Gm20594 is significantly downregulated (DESeq2; p=0.04103). The control had an expression (log2) of 3.20 while the experimental had an expression (log2) of 2.24. **C: *Hapln1 Differential Expression Bar Plot.*** Hapln1 is significantly upregulated (DESeq2; p=0.02654). The control had an expression (log2) of 0.12 while the experimental had an expression (log2) of 0.96. **D:** ***Table of Statistical Tests on Gm20594 and Hapln1.*** Gm20594 and Hapln1 were selected as they were found to be statistically significant in more than one analysis. Their average expression values for the control (AveExprC) and the experimental (AveExprE) groups are also listed.

## Description

It is important to determine what causes adverse health effects in astronauts who have experienced spaceflight, especially considering their exposure to microgravity and cosmic radiation. Microgravity, experienced at low-Earth orbit, is a reduction in the Earth’s gravity leading to weightlessness or near weightlessness (Hicks, et al., 2023). A study by Baran, et al., observes that spaceflight can be associated with cardiovascular risk factors in astronauts who experience altered cell shapes, cardiac atrophy, and endothelial dysfunction (Baran, et al., 2021). A review conducted by Giacinto, et al., points to data obtained from epidemiological studies of those exposed to radiation on Earth, indicating that the cardiovascular system is susceptible to significant damage from ionizing radiation (Giacinto, et al., 2024).

To investigate changes in gene expression resulting from chronic irradiation and simulated microgravity on cardiac tissue, C57BL/6J mice were housed in a space simulation facility at the University of Arkansas for Medical Sciences (UAMS). For 30 days, the mice were exposed to prolonged Cs-137 gamma irradiation. A hind-limb uploading model was used to simulate microgravity (NASA approved). Experimental mice were returned to Earth's gravity with no radiation for an additional 60-day growth phase, allowing for the assessment of the late effects of exposure. Control mice were raised in the same facility but under Earth gravity without irradiation. At the conclusion of the experiment, all mice were euthanized, and cardiac muscle tissue was harvested for RNA sequencing.

Transcriptomic analysis was done using the BigOmics Analytics RNA-Seq platform (BigOmics Analytics LLC, Sweden, Akhmedov et al., 2020). Sample quality control (QC) and principal component analysis (PCA) were used to determine data quality and structure. Because the variability between mice and the small sample size potentially limited our ability to detect significant differences in gene expression, we initially used three different methods for differential gene expression analysis: DESeq2 (Love, Huber, and Anders, 2014), edgeR (Robinson, McCarthy, and Smyth, 2010), and limma (Ritchie et al., 2015). For this preliminary analysis, we only considered genes that were found to be significantly expressed in more than one method. However, we reported results from the DE analysis using DESeq2, as it showed Hapln1 and GM2059 expression, with the lowest significant difference (false discovery rate) ( Figure 1A ).

Hapln1 (Mouse Genome Informatics, 2024) was significantly upregulated in the experimental group when compared to the control group ( Figure 1C ). Its human orthologue, HAPLN1 (NCBI ID: 1404), is associated with hyaluronic acid binding activity and compression resistance (National Library of Medicine, 2025; HAPLN1) of the ECM (Ding, H., et al., 2019). Reduced levels of hyaluronan in the ECM indicates greater inflammation within the tissue resulting from tissue damage or infection (Lee-Sayer, S., et al., 2015). Naïve T cells begin upregulating CD44 (a hyaluronan receptor) as an immune response (Lee-Sayer, S., et al., 2015). This implies that microgravity and radiation induce heart tissue inflammation, necessitating an immune response with an increase of HAPLN1—and hyaluronic acid binding activity—to attempt to reduce the inflammation.

Inflammation of the vascular wall is caused by microgravity-induced oxidative stress on endothelial cells and vascular smooth muscle cells (Han, H., et al., 2024). This results in reduced contractility as the heart wall thickens due to inflammation (Han, H., et al., 2024). Endothelial cells are particularly important for heart development, cell growth, and regulation of vascular tone (Hsieh, P., et al., 2009) while vascular smooth muscle cells perpetuate vascular homeostasis and contractility (Steucke, K., et al., 2016).

Unlike Hapln1, GM20594 expression was significantly decreased in mouse hearts due to simulated space environments. In mice, Gm20594 has been shown to be upregulated in brown adipose tissue (Kim et al., 2021) and has been suspected to protect cells from oxidative stress (Yen et al., 2020). However, GM 20584 was chosen for analysis because it is not well characterized in mice. Interestingly, its human ortholog, MTRNR2L7 (Mouse Genome Informatics, 2024) is also not well characterized, but has gene ontology terms connecting it to the regulation of apoptosis (National Library of Medicine, 2025; NCBI ID: 100288485).

Previous studies have linked cardiovascular disease in astronauts after space flight to microgravity-induced apoptosis of tissue (Delp et al. 2016). A study by Walls (et al.,2021) showed an increase in cardiomyocyte apoptosis and inflammation as a result of prolonged exposure to microgravity in *drosophila* flies. The downregulation of GM20594 (MTRNR217) observed in our study may further exacerbate the effects of oxidative stress-induced apoptosis during spaceflight. However, further work is needed to correlate gene expression with physiological symptoms directly.

The data obtained from our preliminary mouse experiments indicate that genes Gm20594 and Hapln1 are differentially expressed in cardiac muscle when exposed to microgravity and radiation. Although physiological effects were not measured, the downregulation of Gm20594 is consistent with the increased inflammation, contractile dysfunction, and cardiomyocyte apoptosis reported by others (Delp et al. 2016, Prasad, et.al 2020, Walls, et al., 2021). Our experiments begin to link tissue-specific gene expression with the adverse effects associated with long-term space flight travel. For astronauts, this is the first step in developing countermeasures that can mitigate the adverse effects related to long-term spaceflight.

## Methods

**Hindlimb unloading (simulated microgravity) and Gamma Radiation**

Six-month-old male and female C57BL/6J mice were housed in a 175 cm² trapezoidal cages equipped with hind-limb unloading (HLU) mechanisms. Hind limbs were suspended at a 30° angle using a tail harness attached to a swivel buckle mounted to a guide wire, allowing full access to all areas of the cage (Globus and Morey-Holton, 1985; Morey-Holton and Globus, 1998). Cages were arranged at equal distances in a circular formation around a Cs-137 gamma source. After 30 days, the treatment mice received a cumulative dose of 0.5 Gy at a rate of 0.01 Gy/h. After 30 days of exposure (radiation/microgravity), mice were returned to their cages and housed without radiation or microgravity for an additional 60 days before tissue harvest. Control mice were raised in the same facility without hind-limb unloading and shielded from gamma radiation. All animal experiments were approved by the IACUC Committee of the University of Arkansas for Medical Sciences (IACUC- No. 4139), and all procedures were conducted in accordance with relevant guidelines and regulations.

**Tissue Harvest**

Immediately after the animals were euthanized, skeletal muscle tissue was harvested using sterile microsurgical tools and cut into ≤0.5 cm pieces, and each piece was placed in 1 ml of *RNAlater* Stabilization Solution according to the manufacturer’s recommendations (Invitrogen, catalog #AM7020). Samples in RNAlater were transferred to a 4°C refrigerator for 2 days to allow thorough penetration of the solution into the tissue. After 2 days, excess RNAlater solution was removed, and the samples were stored at -80°C until processing for the RNA preparation and sequencing

**RNA Isolation**

Frozen tissue was homogenized in ZR BashingBead Lysis Tubes (Zymo, catalog # S6003) using Bullet Blender 5E Gold (Next Advance). Total RNA was extracted from the homogenized tissue using Quick-DNA/RNA ^TM^ Miniprep Plus Kit (Zymo Research, catalog # D7003) as described in the manufacturer’s instructions. The final RNA concentration was measured by Qubit™ RNA BR Assay (Thermo Fisher Scientific, catalog # Q10210). RNA quality was assessed by Fragment Analyzer System (Agilent) using RNA kit (15 nt) (Agilent, catalog # 5191-6572) ( *Schroeder A et al., 2006* ). DV200 values representing the percentage of RNA fragments above 200 bp in length were determined as described previously ( *Matsubara et al., 2020* )

**RNA Sequencing** .

1 µg of total RNA was used as input to generate sequencing libraries using TrueSeq Stranded Total RNA Library Prep Gold Kit (Illumina, catalog # 20020599) following the manufacturer’s protocol in the reference guide. Briefly, total RNA was rRNA-depleted, fragmented, and reverse transcribed into cDNA. Then, double-stranded cDNA libraries were prepared by A-tailing adaptor ligation and index PCR amplification. The concentration of final libraries was assessed by Qubit™ 1X dsDNA HS Assay (Thermo Fisher Scientific, catalog # Q33231). The quality control of libraries was performed on the Fragment Analyzer System (Agilent) using HS NGS Fragment kit (1-6000 bp) (Agilent, catalog # 5191-6578), and QuantStudio Real-Time PCR system using KAPA Library Quantification Kit (Kapa Biosystems, catalog # KK4824). Libraries were sequenced either on a NextSeq2000 or NovaSeq 6000 (Illumina) with paired-end mode (read1: 101 cycles, read 2: 101 cycles, i7: 8 cycles, i5: 8 cycles)

**RNA-sequencing Bioinformatics Analysis**

RNA reads were checked for quality of sequencing using FastQC, v0.11.8. The adaptors and low-quality bases (Q < 20) were trimmed to a minimum of 36 base pairs using Trimmomatic, v0.39 (Bolger, Lohse and Usadel, 2014). Reads that passed quality control were aligned to the reference genome, *Mus musculus* GRCm39.104, using STAR, version 2.7.1a (Dobin et al., 2013). Raw counts were obtained from BAM files using Subread's features Count function (vs 2.0.0).

RNA sequencing (RNA-seq) count data were analyzed using the BigOmics Analytics platform, a web-based bioinformatics tool designed for interactive data exploration and differential expression analysis. The raw RNA-seq counts underwent filtering to remove lowly expressed genes. Genes were excluded if they did not meet a minimum count threshold across a sufficient number of samples. To account for differences in sequencing depth and RNA composition across samples, the data were normalized using the method appropriate for each statistical framework. The counts were normalized based on the median-of-ratios for DESeq2, edgeR applied the trimmed mean of M-values (TMM) normalization method, and limma used the vroom transformation to model the mean-variance relationship and produce precision weights for linear modeling.

Differential expression was assessed using three parallel statistical frameworks to ensure robust and cross-validated results. DESeq2 is a model based on a negative binomial distribution fitted to the count data, where dispersion estimates are moderated across genes, and the likelihood ratio test was applied. edgeR relies on negative binomial modeling with empirical Bayes moderation of dispersion estimates. Exact tests were used for pairwise comparisons. Limma-voom employed linear models fitted to the log-counts per million (log-CPM) data. Empirical Bayes moderation is also applied to improve the variance estimates across genes. Each method provided log2 fold change estimates and associated p-values, which were adjusted for multiple testing using the Benjamini-Hochberg false discovery rate (FDR). Genes with FDR-adjusted p-value < 0.05 and absolute fold change > 2 were considered significant.

BigOmics Analytics provided interactive visualizations, including principal component analysis (PCA) and volcano plots, to facilitate quality control and interpretation of results. The use of multiple statistical methods enabled users to cross-validate the results.
